# MAP/Microtubule Affinity Regulating Kinase 4 Inhibitory Potential of Irisin: A New Therapeutic Strategy to Combat Cancer and Alzheimer’s Disease

**DOI:** 10.3390/ijms222010986

**Published:** 2021-10-12

**Authors:** Rashid Waseem, Saleha Anwar, Shama Khan, Anas Shamsi, Md. Imtaiyaz Hassan, Farah Anjum, Alaa Shafie, Asimul Islam, Dharmendra Kumar Yadav

**Affiliations:** 1 Centre for Interdisciplinary Research in Basic Sciences, Jamia Millia Islamia, Jamia Nagar, New Delhi 110025, India; rashid.waseem439@gmail.com (R.W.); email2saleha@gmail.com (S.A.); anas.shamsi18@gmail.com (A.S.); mihassan@jmi.ac.in (M.I.H.); 2 Drug Discovery and Development Centre (H3D), University of Cape Town, Rondebosch 7701, South Africa; shamak361@gmail.com; 3 Department of Clinical Laboratory Sciences, College of Applied Medical Sciences, Taif University, P.O. Box 11099, Taif 21944, Saudi Arabia; f2016anjum@gmail.com (F.A.); dr.alaa.shafie.tu@gmail.com (A.S.); 4 College of Pharmacy, Gachon University of Medicine and Science, Hambakmoeiro, Yeonsu-gu, Incheon City 21924, Korea

**Keywords:** protein-protein interaction, kinase inhibitors, cancer therapy, neurodegenerative diseases, molecular dynamics simulation, microtubule dynamics

## Abstract

Irisin is a clinically significant protein playing a valuable role in regulating various diseases. Irisin attenuates synaptic and memory dysfunction, highlighting its importance in Alzheimer’s disease. On the other hand, Microtubule Affinity Regulating Kinase 4 (MARK4) is associated with various cancer types, uncontrolled neuronal migrations, and disrupted microtubule dynamics. In addition, MARK4 has been explored as a potential drug target for cancer and Alzheimer’s disease therapy. Here, we studied the binding and subsequent inhibition of MARK4 by irisin. Irisin binds to MARK4 with an admirable affinity (*K* = 0.8 × 10^7^ M^−1^), subsequently inhibiting its activity (IC_50_ = 2.71 µm). In vitro studies were further validated by docking and simulations. Molecular docking revealed several hydrogen bonds between irisin and MARK4, including critical residues, Lys38, Val40, and Ser134. Furthermore, the molecular dynamic simulation showed that the binding of irisin resulted in enhanced stability of MARK4. This study provides a rationale to use irisin as a therapeutic agent to treat MARK4-associated diseases.

## 1. Introduction

Irisin is a recently identified myokine, a cleaved fibronectin type III domain containing 5 (FNDC5) and secreted into blood circulation upon physical exercise. It was found to stimulate adipose tissue browning and regulate thermogenesis [1,2]. Irisin is widely expressed in skeletal muscles, heart, brain, liver, and salivary glands [1,3]. Recent studies have reported various other beneficial effects of irisin, including the regulation of depression [4], insulin sensitivity [5], glucose homeostasis [6], osteoblast proliferation [7], and neurogenesis [8], highlighting the importance of irisin. Instead of these irisin effects, it can be a potent drug target for many diseases ranging from obesity to dementia. According to a recently published piece of literature, irisin stimulates brain-derived neurotrophic factor (BDNF) in the brain’s hippocampal region and plays an important role in brain function [9].

Moreover, irisin was suggested to be involved in the prevention of Alzheimer’s disease (AD). Irisin modulates various AD risk factors and shows protective action against the abnormal expression of synapse-related genes, which can attenuate the synaptic and memory dysfunction in AD models [10]. All these studies imply that irisin is a potential therapeutic agent for neurodegenerative disorders. Hence, pharmacologically, boosting brain irisin levels can be a therapeutic strategy to protect/repair synapse function, thereby preventing cognitive decline in AD.

One of the pathological hallmarks of AD is the accumulation of the microtubule-associated protein tau and hyper-phosphorylation of tau in the brain that is intricately involved in the progression of AD. Overexpression of different kinases is related to multiple cancers and neurodegenerative diseases. MAP (Mitogen-activated protein kinase)/Microtubule Affinity Regulating Kinase 4 (MARK4) is a Ser-Thr kinase that plays a key role in early tau phosphorylation, leading to Alzheimer’s disease [11]. MARK4 has an important role in AD progression. It phosphorylates the tau at the Ser^262^ site, resulting in the detachment of tau from microtubules, thus allowing it to be phosphorylated by other kinases [12]. Numerous reports have suggested MARK4 as one of the best targets for AD therapy and other neurodegenerative diseases [13,14]. Due to the various important roles of MARK4 in neurodegenerative diseases, MARK4 is considered an attractive drug target; therefore, its inhibition can be a therapeutic strategy for treating neurodegenerative disorders [15,16,17].

For pharmacological profiling, it is important to investigate the interaction mechanisms of therapeutic proteins or drugs with target proteins [18,19,20]. The pharmacokinetic properties of therapeutic proteins or drugs depend upon their interaction with target proteins or tissues [21,22,23,24]. It is essential to study the protein-protein or protein-drug interaction to compete with pharmaceutical industry advancements [25,26,27,28]. Protein-based therapeutics are very successful and have high clinical significance [29,30]. More than 100 genuine and modified therapeutic proteins are approved for clinical use in the European Union and the USA [31]. To date, a vast number of drugs have been designed against the targeted proteins [32,33,34]. Naturally derived and chemically synthesize inhibitors are used effectively against the targeted proteins [35,36,37,38,39,40,41,42,43]. Irisin has a vital role in various metabolic and neurological problems, indicating its high therapeutic value, so using irisin as a therapeutic agent can be a good strategy for pharmaceutical industries. 

For the first time, this study gives a rationale for targeting the inhibition of MARK4 with irisin, a protein–protein interaction. This approach might provide us a platform to use irisin as a therapeutic agent in targeting different kinases that are overexpressed in various diseased conditions [44]. In this study, we have expressed and purified irisin and MARK4 using Ni-NTA chromatography. Fluorescence-based binding and isothermal titration calorimetry (ITC) ascertained the actual binding of irisin with MARK4. Molecular docking and simulation were performed to validate the in vitro observations, and these suggested that the binding of irisin increases the stability of MARK4.

## 2. Materials and Methods

### 2.1. Materials

The expression construct of irisin (pET15b-His-3C-Irisin) was purchased from Addgene (122612). Unless stated, all the chemicals were purchased from Sigma-Aldrich Co. (St. Louis, MO, USA). Other analytical grade reagents were procured from local suppliers.

### 2.2. Expression and Purification of Irisin and MARK4

Human irisin and MARK4 proteins were expressed and purified as per our published protocol, respectively [3,45]. Kinase assay, SDS-PAGE investigated the quality of the purified recombinant MARK4. The MARK4 protein was further confirmed with Western blot using peptide-specific primary antibodies [46].

### 2.3. Fluorescence Measurements

For studying the binding affinity of irisin with MARK4, the fluorescence emission spectrum was recorded using the Jasco spectrofluorometer (FP-6200) and analyzed as per previous studies [3,47]. Fluorescence quenching data were fitted into a modified Stern−Volmer equation to estimate the different binding parameters of MARK4–irisin interaction.

### 2.4. Isothermal Titration Calorimetry

ITC measurements were carried out on a VP-ITC microcalorimeter from MicroCal, Inc (MicroCal, Amherst, MA, USA) as previous reports [30,47]. The first injection was a false one of 2 s with other injections set at 10 s. Before loading, degassing was carried out to remove the bubble hindrance. The sample cell was filled with 15 µM MARK4, and the syringe was loaded with 200 µM irisin. The final figure was obtained with the attached software (MicroCal 8.0), and the data was plotted as a two-site model.

### 2.5. Kinase Assay

An ATPase assay was conducted to check the inhibitory effect of varying irisin concentrations on MARK4 as previously published protocols [48,49].

### 2.6. Protein Structure Modeling and Preparation

The high-resolution crystal structures of human MARK4 and irisin were retrieved from the Protein Data Bank [50]. We used atomic coordinates of the PDB code of 5ES1 (https://www.rcsb.org/structure/5es1, accessed on 10 September 2021) for MARK4 (2.80 Å resolution) for molecular docking residue numbers 51–366. The structure was further refined, and all missing residues were added. We used the structure of MARK4 since it mainly consists of kinase and UBA domains. We aimed to see the kinase inhibitory potential of irisin; therefore, our focus was on the kinase domain. We have cloned, expressed, and purified the recombinant MARK4 containing both kinase and UBA domains as described [51,52].

The atomic coordinates of the irisin structure were downloaded from the PDB (https://www.rcsb.org/structure/4LSD, accessed on 10 September 2021). The 2.28 Å resolution structure of irisin contains residues 30–126. Irisin is produced by proteolytic processing of a transmembrane receptor, fibronectin domain-containing protein 5 (FNDC5), which is comprised of an N-terminal 29-residue signal sequence, followed by the irisin domain or fibronectin III (FN III)2 domain, a transmembrane domain, and a 39-residue cytoplasmic segment [1]. Thus, we cloned the mature peptide of irisin consisting of amino acid residues 30–140 only for experimental studies.

The structure of MARK4 was pre-processed, minimized, and refined using the Protein Preparation Wizard implemented in the Schrödinger suite [53]. This correlated by removing crystallographic waters and adding missing side chain or hydrogens atoms, with an accurate charge and protonation state assigned to the enzyme structure persistent to neutral pH (7.0) while recognizing the appropriate ionization states for the basic as well as acidic amino acid residues. The structure was then exposed to energy minimization using the OPLS-2005 force-field [54] with an RMSD cut-off value of 0.30 Å to reduce the steric clashes between the residues due to the addition of hydrogen atoms. A standard proceedure of docking was implicated as described [55,56,57].

### 2.7. Protein–Protein Docking

Molecular docking was performed using the PIPER program implemented in the BioLuminae module of Schrodinger’s protein–protein docking workflow to identify the best binding scores relevant for the MARK4 enzyme [58]. It is an effective program that removes false-positive poses and executes a comprehensive search with a fast Fourier transform (FFT) approach. Using 100 conformations of input structures, the top 20 clusters were selected with cluster radius 10 Å, which then centralized, and the docking results based on cluster size were assessed. The docked complex out of 5 complexes was studied for molecular dynamics simulation with a large cluster size.

### 2.8. Molecular Dynamics Simulations

Molecular dynamics (MD) simulation has served as an essential tool for comprehending macromolecules’ structural, functional and dynamic behavior [59]. GPU accelerated simulation engine PMEMD programmed in the Amber 18 package implemented MD simulations on two prepared systems [60]. FF14SB force-field [61] was used to describe the enzyme. Both systems were fully solvated in a TIP3P computer-generated water box [62] inside 10 Å box edge with LEaP component [63] of Amber 18. We have added Na^+^ and Cl^−^ counter ions for neutralizing the system leading to the minimization phase with the same LEaP component of Amber 18. A two-step—partial and full minimization of 1500 and 1000 steps were achieved with a 500 kcal/mol restraint potential and conjugate gradient method followed by eliminating the restraints. Systems were then heated from 0 K to 300 K for 50 ps. Later, equilibration was accomplished by retaining the 500 ps equilibration step ensuring 300 K firm temperature. To maintain a steady atom numbers of and pressure for each system, NPT (isobaric-isothermal ensemble) was permitted. Berendsen barostat was applied to preserve the pressure at 1 bar on both systems [64]. Finally, a 100 ns MD simulations run was performed on both systems by incorporating the SHAKE algorithm [65] to control the hydrogen bond atoms. Details of MD simulation method has been described elsewhere [66].

### 2.9. Post-Dynamic Trajectories Analysis

Apo and MARK4-Myricetin complex were stored after every 1 ps, and the CPPTRAJ component [67] incorporated into Amber 18 package was used to analyze the MD trajectory curves. The RMSD We have assessed RMSF *R*_g_, SASA, formation of intramolecular hydrogen bonds, distance correlation matrix with the secondary structure on the final MD trajectories. For scheming and analysis of these MD trajectories, Origin [68] and VMD visualization tools [69] were utilized. 

### 2.10. Dynamic of the Cross-Correlation Matrix

The DCCM was assessed using the CPPTRAJ module employed in Amber 18 and all the matrices schemes using Origin software as described earleir [70]. Factors for i and j cross-correlation Cα atoms are shown underneath:

(1) $$C_{ij}\frac{<\Delta ri.\Delta rj>}{{\left(<\Delta r_{i}^{2}><\Delta r_{j}^{2}>\right)}^{\frac{1}{2}}}$$

where Δr_i,j_ is the movement of the *i*th and *j*th atom average point and the angle braces are specified over the complete curves. All correlated movements are symbolized by C_ij_ = 1; however, C_ij_ = −1 denoted highly anti-correlated movements over time. The deviation of movements from 1 to −1 describes that i and j movements are correlated and anti-correlated, respectively. The DCCM was assessed using the CPPTRAJ module employed in Amber 18 and all the matrices schemes using Origin software.

### 2.11. Binding Energy Calculation

PRODIGY (PROtein binDIng enerGY) [58] is a freely available web server used to evaluate the binding free energy (ΔG) and to predict the dissociation constant (*K*_d_) of the protein-protein complex. This server implements a highly effective predictive model based on intermolecular contacts and properties derived from the non-interface surface.

## 3. Result and Discussion

### 3.1. Fluorescence-Based Binding Analysis

**Figure 1:**
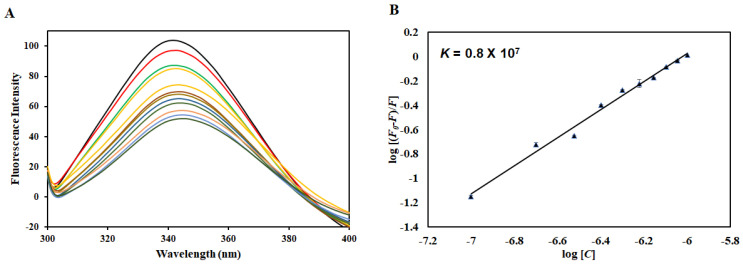
(**A**) Intrinsic fluorescence of MARK4 in the absence and presence of irisin (0–1 µM). (**B**) Modified Stern–Volmer plot of MARK4–irisin interaction to find binding constant.

Human irisin and MARK4 proteins were successfully purified as per previously published reports [3,45]. Fluorescence-based binding is a commonly deployed technique retorted to analyze the actual interaction between protein and ligand [71]. With the use of intrinsic fluorescence, changes in the local microenvironment of aromatic amino acid residues are investigated [72,73]. In fluorescence quenching, a decrease in fluorescence intensity is evident with increasing ligand concentration suggesting the formation of the complex [74]. Native MARK4 shows a fluorescence emission spectrum at 344 nm, characteristic of the native protein. However, with increasing irisin concentration (0–1 µM), a decline in the fluorescence intensity was apparent implicative of the complex formation between MARK4 and irisin (Figure 1A). This decrease in the fluorescence was analyzed employing the MSV equation as per previously published reports [48,70].

Figure 1B shows the experimental fitting obtained according to this equation, with the slope of the plot giving the number of binding sites (*n*) and the intercept giving the binding constant (*K*). Irisin binds to MARK4 with an excellent binding affinity evident from the obtained binding constant (*K* = 0.8 × 10^7^ M^−1^). This analysis reveals that irisin binds to MARK4 with an excellent affinity and can serve as a potent binding partner of MARK4 that can be implicated in the management of diseases in which MARK4 is overexpressed, contributing to the development of diseases viz. various cancers and neurodegenerative diseases such as AD. 

### 3.2. Isothermal Titration Calorimetry

**Figure 2:**
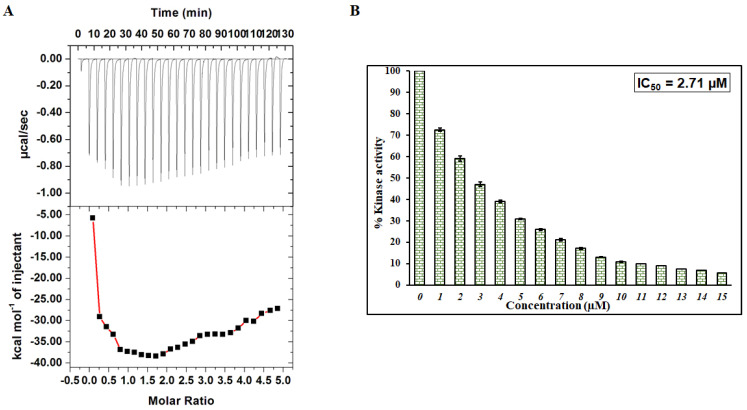
(**A**) ITC profile of MARK4-irisin system. The sample cell was filled with 15 µM MARK4 while the syringe contained 200 µM irisin. (**B**) Kinase inhibition assay of MARK4 with varying irisin concentration (0−15 µM).

ITC is an affirmative technique deployed to complement the binding studies [30]. Thus, to validate earlier observations, ITC was performed to understand the binding parameters of MARK4-irisin interaction and find its associated thermodynamic parameters [75]. Figure 2A depicts a typical isotherm obtained upon titration of 200 µM irisin with 15 µM MARK4. The obtained isotherm for MARK4-irisin interaction suggests that irisin is binding spontaneously to MARK4 and these observations are in line with earlier results affirming strong binding between MARK4 and irisin. The upper panel shows the raw data owing to consecutive irisin injections into MARK4. In contrast, the bottom panel depicts the binding curves obtained after subtracting the dilution heat of the MARK4 and irisin. Table 1 depicts various thermodynamic parameters obtained for MARK4-irisin interaction, and the results presented were obtained from two-site model fitting.

### 3.3. Kinase Assay

In vitro and in silico observations, it is evident that irisin binds to MARK4 with an excellent affinity. Thus, it becomes imperative to evaluate the kinase inhibitory potential of irisin. MARK4 in the absence of irisin was taken as 100% for reference. Figure 2B shows the effect of varying concentrations of irisin on the kinase activity of MARK4, suggesting that when irisin concentration increases, there is a corresponding decrease in the activity of MARK decreases. IC_50_ is that ligand concentration where 50% inhibitory effect is observed. According to earlier published studies [48,76] it was calculated as 2.71 µM making use of AAT Bioquest calculator. IC_50_ of such order implies irisin to be a potent MARK4 inhibitor and corroborates with earlier observations.

### 3.4. Molecular Docking

**Figure 3:**
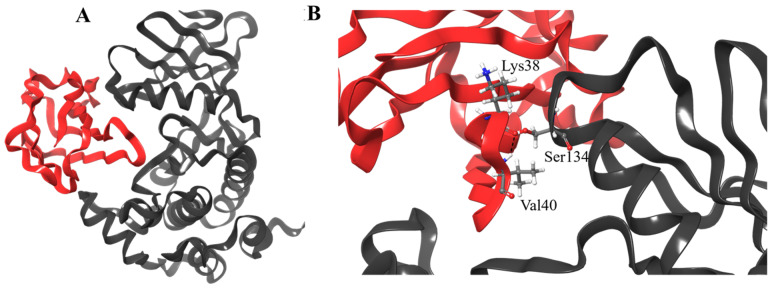
(**A**) Structural representation of MARK4 enzyme (ribbon view—black color) in complex with irisin (ribbon view—red color). (**B**) Close view of interactions formed between the carbon backbone atoms of MARK4 and irisin.

**Figure 4:**
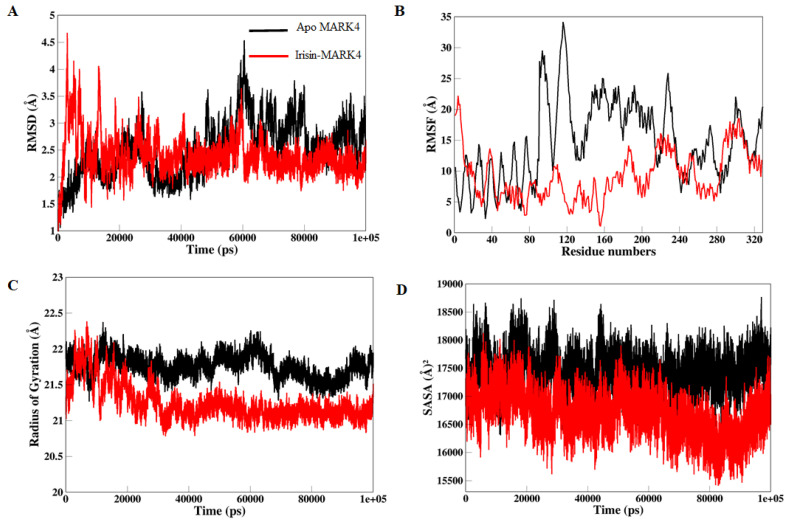
Structural dynamics of MARK4 apo (black color) and irisin bound MARK4 (black color) enzyme. (**A**) RMSD, (**B**) RMSF, (**C**) R_g_ values, and (**D**) SASA values across Cα backbone in Å of apo MARK4 and irisin-MARK4 complex in Å across Cα backbone of both of the two conditions calculated after 100 ns of MD trajectories.

The three-dimensional structure of irisin was retrieved from the PDB database as stated before, to assess the potential interaction within the binding site of MARK4 protein by the molecular docking approach (Figure 3). We achieved an optimized positioning of the irisin chain in the protein’s binding site by minimizing the comprehensive energies of the corresponding complex. The predictable value of the binding affinity of irisin was −10.0 kcal/mol. As shown in Figure 4B, the protein–protein complex of irisin and MARK4 proteins has been formed by multiple hydrogen bonds through the active site residues Lys38, Val40, and Ser134 of the irisin-MARK4 complex. On the other hand, the predicted dissociation constant was reported as 4.8 × 10^−8^ M.

### 3.5. Post-Dynamics Trajectories Analysis

Protein-protein interactions manage a broad range of biological activities, involving cell-to-cell interfaces, metabolic and developmental influence [77]. Differences within protein structure are explicitly correlated with their genomic behaviors [78]. The inhibitory activity of many enzymes convoluted in various infectious pathways is extremely affected upon the binding of small molecules. Hence there is necessary to consider the conformational changes and structural dynamics correlated with the inhibitory activity of these complexes [79]. To determine the efficiency and consistency of the simulated MARK4 protein in complex with Irisin, we have estimated the time-variability through RMSD of Cα atoms from constructed trajectories. As shown in Figure 4A the RMSD perturbed values uncovered possible symmetrical deviations in protein structure after binding of studied protein. Apo MARK4 and Irisin-MARK4 complex were stabilized and achieved convergence after 40 ns of the simulation time as indicated in the 2D plot displayed in Figure 4A. The least RMSD value of 2.57 Å was observed in the Irisin-MARK4 complex, while apo MARK4 suggested an average of 3.25 Å separately. Apo MARK4 protein revealed high fluctuations in contrast with the bound MARK4 complex. This RMSD assessment suggests that the irisin-MARK4 complex showed the least deviation of Cα backbone atoms, indicating that binding of this employed irisin protein boosted stability on MARK4 protein comparative to apo MARK4. The RMSD evaluation also proposes that any further reports on the determined trajectories for the apo and irisin-MARK4 complex were reliable.

The biological activity of the target protein is supported by the rigidity and flexibility of its amino acid residues and biological activity in several biological pathways [80]. RMSF of Cα atoms were evaluated from the generated trajectories during 100 ns of MD simulations to explain the flexibility and rigidity of overall MARK4 residues upon irisin binding. The Irisin-MARK4 complex suggested the least fluctuations in the amino acid residues with an average RMSF value of 9.93 Å as shown in Figure 4B. Apo MARK4 protein displayed a significantly higher RMSF value of 18.36 Å. The RMSF value of Irisin-MARK4 is notably lower than apo MARK4, disclosing a substantial activity of the Irisin-MARK4 complex against apo MARK4. The residues from 80–240 revealed high fluctuations throughout the MD simulation time as this region contributes to the binding of Irisin to MARK4 protein. The effective binding of this Irisin protein chain in the active site might be connected with the structural inactivation of MARK4 protein and have a more complex structural effect on various parts of the overall MARK4 structure.

Furthermore, to be consistent with our findings, we have assessed radius of gyration (*R*_g_) values, a factor associated with the overall conformational improvements within protein structure after Irisin binding. It also uncovers protein’s structural compactness, folding behavior and stability [66]. We calculated the *R*_g_ values to study the compactness of Irisin-MARK4 and apo MARK4 protein. As shown in Figure 4C, a notable increase was seen in the Rg value of apo MARK4 protein, whereas the Irisin-MARK4 complex showed a least *R*_g_ value of 21.14 Å. Apo MARK4 protein uncovered the elevated *R*_g_ value of 22.24 Å in comparison with the Irisin-MARK4 complex. This estimate suggested improved compactness and increased activity of Irisin upon MARK4 binding. The stability, flexibility, and efficiency of irisin with MARK4 protein indicate positive discoveries associated with its conformational dynamics.

Solvent accessible surface area (SASA) was measured after studying the conformational binding studies to describe the efficiency of hydrophobic and hydrophilic amino acid residues and energies subjected to the solvent throughout the simulation time [81]. The enzyme inhibition usually affects by influences between the hydrophobic intrinsic contacts inside the protein structure. These hydrophobic networks generated between the non-polar amino acid residues justify the protein structure’s stability inside the solution by defending the non-polar residues in the hydrophobic core isolated from an aqueous mixture [82]. For the Irisin-MARK4 complex and apo MARK4 protein, total SASA values were assessed throughout the 100 ns MD simulation production period (Figure 4D). We exposed the Irisin-MARK4 complex and apo MARK4 protein to the solvent and obtained the standard SASA value of 16,517 Å^2^ in the Irisin-MARK4 complex leading to an increased average SASA value of 17,874 Å^2^ was noted in apo MARK4 protein as compared to the Irisin-MARK4 complex. An improved SASA value was revealed in Irisin-MARK4 against apo MARK4 protein. The folding and unfolding behavior of the MARK4 protein was marked through the modifications in SASA values for the Irisin-MARK4 complex during this simulation time. All these predictions favored the improved exposure of irisin to solvent and also indicated its enhanced inhibitory activity over apo MARK4 protein.

### 3.6. Hydrogen Bond Analysis

**Figure 5:**
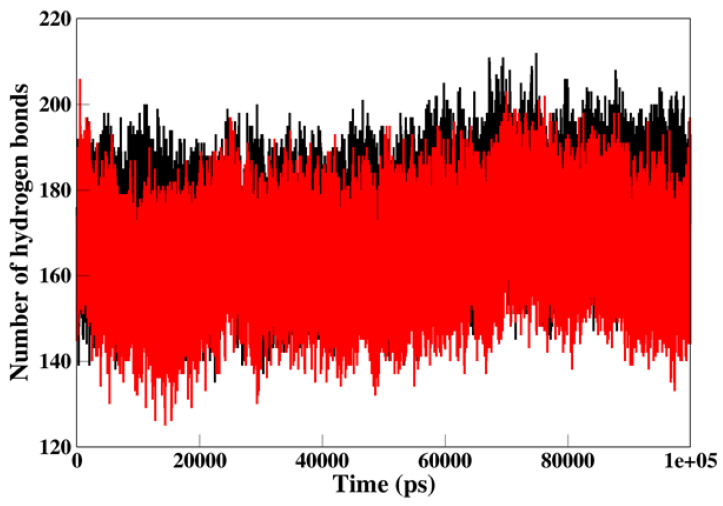
Hydrogen bond analysis. Intramolecular hydrogen bonds in apo (black color) and irisin-MARK4 complex (red color) were calculated after 100 ns MD simulation.

Intramolecular hydrogen bond assessment helps in estimating the stability of the protein-ligand complex (Figure 5). This analysis offers an overall understanding of the mechanism involved in protein-protein binding. The number of intramolecular hydrogen bonds in the Irisin-MARK4 complex was approximately 125–180, whereas 145–190 in the apo MARK4 protein, respectively. Apo MARK4 protein indicated the least number of intramolecular hydrogen bonds formation in comparison to the Irisin-MARK4 complex.

### 3.7. Distance Correlation Matrix

**Figure 6:**
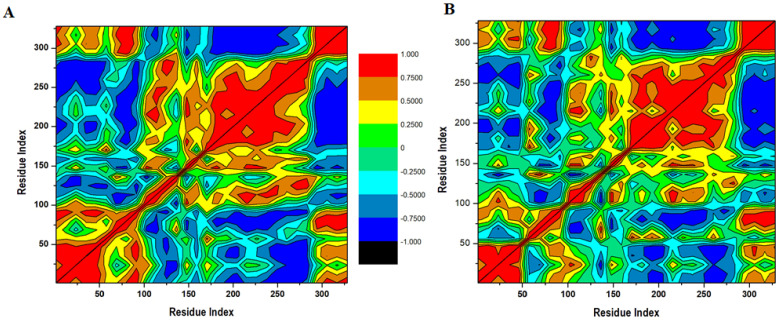
Dynamics cross-correlation matrix analysis (**A**) apo MARK4 enzyme and (**B**) irisin-MARK4 complex calculated after 100 ns of MD trajectories.

A distance correlation matrix was analyzed for the apo MARK4 and Irisin-MARK4 complex for the correlated and anti-correlated developments of all the amino acid residues, as displayed in Figure 6. Various residual movements were observed and MARK4 protein was scattered into several communities across positive and negative correlations. More negative and less positive residual movements were seen in apo MARK4 protein during the whole 100 ns MD simulation run (Figure 6A). On the other side, the Irisin-MARK4 complex produced a significant positive correlation of motions in the amino acid residues (Figure 6B). The residues between 150–270 in apo MARK protein contributed to the most negative correlation indicative of no inhibition without Irisin (Figure 6A). On the other side, the residues between 1–50 and 160–280 contributed to positive developments in the Irisin-MARK4 complex, indicative of improved Irisin activity to MARK4 protein.

### 3.8. Secondary Structure Analysis

**Figure 7:**
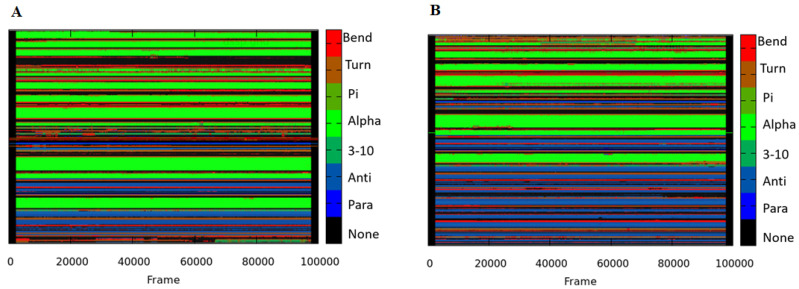
Secondary structure analysis (**A**) apo-MARK4 enzyme and (**B**) irisin-MARK4 complex calculated after 100 ns of MD trajectories.

We also measured the secondary structure modifications for the apo and Irisin-MARK4 complex in favor of all the above-mentioned findings. This analysis serves as an important tool to observe the changes in the MARK4 structure in the absence and presence of Irisin. The 2D plots for apo and Irisin-MARK4 complex are displayed in Figure 7. The structural components are higher in the Irisin-MARK4 complex in contract with apo MARK4 protein, as shown in Table 2. The number of α-helix in apo MARK4 protein is slightly increased, whereas the β-strands and 3_10_-helix contributed majorly in the Irisin-MARK4 complex. The strands and helices in the Irisin bound protein indicate improved activity of MARK4 protein after Irisin binding in the active site of this protein. Strong stability and flexibility of Irisin residues to MARK4 protein was revealed through this secondary structure analysis that could indicate Irisin importance to MARK4 protein.

## 4. Conclusions

The present study establishes irisin as an inhibitor of MARK4, highlighting its role in cancer and AD therapy. This work reveals the therapeutic aspect of irisin via its binding to MARK4 with an excellent affinity and subsequent inhibition of kinase activity. Overexpression of MARK4 is associated with cancers and neurodegenerative diseases. Many studies have reported MARK4 inhibitors that can be retorted in the management of MARK4-associated diseases. For the first time, this study reports the inhibition of MARK4 by irisin, an exercise-induced myokine, opening a new avenue in the therapeutics to the use of irisin as a potential therapeutic agent for targeting different kinases. Thus, pharmacologically or through exercise-induced irisin levels, there can be a therapeutic strategy to treat MARK4–directed cancers and neurodegenerative diseases. 

## Figures and Tables

**Table 1:** Thermodynamic parameters of MARK4–irisin interaction obtained from ITC.

*K*_a_ (Association Constant) M^−1^	∆*H* (Enthalpy Change) cal/mol	∆*S* (cal/mol/deg)
*K*_a1_ = 2.59 × 10^3^ ± 2.10 × 10^4^	∆*H*_1_ = −1.549 × 10^6^ 7617 ± 6.75 × 10^7^	∆*S*_1_ *=* −5.18 × 10^3^
*K*_a2_ = 1.19 × 10^6^ ± 9.73	∆*H*_2_ = 1.898 × 10^5^ ± 1.04 × 10^6^	∆*S*_2_ *=* 664

**Table 2:** Percentage of residues contributing to the secondary structure of apo MARK4 enzyme and irisin-MARK4 complex calculated after 100 ns of MD trajectories.

Protein–Protein Complex	α	β	3_10_-Helix	Turn	Bend	Other
Apo MARK4	28	25	4	13	7	21
Irisin-MARK4	26	27	6	16	11	23

## Data Availability

Not applicable.
